# PCL insufficient patients with increased translational and rotational passive knee joint laxity have no increased range of anterior–posterior and rotational tibiofemoral motion during level walking

**DOI:** 10.1038/s41598-022-17328-3

**Published:** 2022-08-02

**Authors:** Stephan Oehme, Philippe Moewis, Heide Boeth, Benjamin Bartek, Annika Lippert, Christoph von Tycowicz, Rainald Ehrig, Georg N. Duda, Tobias Jung

**Affiliations:** 1 Center for Musculoskeletal Surgery, Charité - Universitätsmedizin Berlin, Berlin, Germany; 2 Berlin Institute of Health at Charité, Universitätsmedizin Berlin, Julius Wolff Institute, Berlin, Germany; 3 Zuse Institute Berlin, Berlin, Germany

**Keywords:** Ligaments, Trauma

## Abstract

Passive translational tibiofemoral laxity has been extensively examined in posterior cruciate ligament (PCL) insufficient patients and belongs to the standard clinical assessment. However, objective measurements of passive rotational knee laxity, as well as range of tibiofemoral motion during active movements, are both not well understood. None of these are currently quantified in clinical evaluations of patients with PCL insufficiency. The objective of this study was to quantify passive translational and rotational knee laxity as well as range of anterior–posterior and rotational tibiofemoral motion during level walking in a PCL insufficient patient cohort as a basis for any later clinical evaluation and therapy. The laxity of 9 patient knees with isolated PCL insufficiency or additionally posterolateral corner (PLC) insufficiency (8 males, 1 female, age 36.78 ± 7.46 years) were analysed and compared to the contralateral (CL) knees. A rotometer device with a C-arm fluoroscope was used to assess the passive tibiofemoral rotational laxity while stress radiography was used to evaluate passive translational tibiofemoral laxity. Functional gait analysis was used to examine the range of anterior–posterior and rotational tibiofemoral motion during level walking. Passive translational laxity was significantly increased in PCL insufficient knees in comparison to the CL sides (15.5 ± 5.9 mm vs. 3.7 ± 1.9 mm, *p* < 0.01). Also, passive rotational laxity was significantly higher compared to the CL knees (26.1 ± 8.2° vs. 20.6 ± 5.6° at 90° knee flexion, *p* < 0.01; 19.0 ± 6.9° vs. 15.5 ± 5.9° at 60° knee flexion, *p* = 0.04). No significant differences were observed for the rotational (16.3 ± 3.7° vs. 15.2 ± 3.6°, *p* = 0.43) and translational (17.0 ± 5.4 mm vs. 16.1 ± 2.8 mm, *p* = 0.55) range of anterior–posterior and rotational tibiofemoral motion during level walking conditions for PCL insufficient knees compared to CL knees respectively. The present study illustrates that patients with PCL insufficiency show a substantial increased passive tibiofemoral laxity, not only in tibiofemoral translation but also in tibiofemoral rotation. Our data indicate that this increased passive multiplanar knee joint laxity can be widely compensated during level walking. Further studies should investigate progressive changes in knee joint laxity and kinematics post PCL injury and reconstruction to judge the individual need for therapy and effects of physiotherapy such as quadriceps force training on gait patterns in PCL insufficient patients.

## Introduction

The posterior cruciate ligament (PCL) is the strongest ligament in the knee joint with a reported tear strength averaging 739-1627N^1,2^. It is the primary stabilizer against dorsal displacement of the tibia relative to the femur. Together with the posterolateral corner (PLC) it inhibits excessive axial external rotation as well^3–7^.

Injuries involving the PCL are severe knee injuries and are associated with pain and chronic instability^8^. The literature varies regarding the incidence of PCL injuries in all knee injuries with numbers ranging from 1 to 44%, depending on the analysed patient cohort and the definition as isolated or combined PCL injury^9–12^. Patients with a PCL insufficiency resulting from a PCL Injury have a significantly higher risk of developing osteoarthritis^13^. Instability due to increased laxity of a joint contributes to the development of osteoarthritis^14^. Restoration of joint instability leads to delayed onset of osteoarthritis^15^, which makes an objective measurement of knee joint laxity in PCL insufficient patients mandatory. The gold standard to diagnose and assess PCL insufficiencies is the quantification of the passive anterior–posterior (AP) translation measured via stress radiography^16^, which leaves the rotational laxity to a secondary status. It is only assessed in the clinical practice by subjective clinical tests, such as the dial test^17^. Although a high degree of translational laxity has been shown in PCL insufficient subjects during passive conditions, a reduced feeling of instability has been reported by patients during active conditions, possibly related to loading dependent kinematical changes in the affected knee^18^. Such kinematical changes could be however related to muscular compensation of excessive motion or to additional risk factors leading to further cartilage degeneration^18,19^.

Despite such evidence, objective measurements of knee joint kinematics during active movements are currently not performed in the clinical evaluation of PCL insufficient patients. Only a few in-silico^20^ and in-vivo analyses^18^ have been conducted to assess knee joint kinematics in PCL insufficient patients during active conditions. Most of them have focused on the translational component^21–26^. The rotational component of PCL insufficiency has been mainly analysed during passive in-vitro assessment in cadaveric studies^3–7,27,28^. In-vivo studies that quantify the passive tibiofemoral rotational laxity of PCL insufficient knees are lacking. The range of rotational tibiofemoral motion of PCL insufficient patients during level walking was evaluated in a few studies, but without quantifying the passive tibiofemoral rotational laxity of the patients^18,22,26^.

The objective of this study was to give a multidimensional biomechanical assessment of knee kinematics in a PCL insufficient patient cohort throughout active and passive conditions. Therefore, we analysed the tibiofemoral rotational and translational laxity in the passive state and range of rotational and anterior–posterior tibiofemoral motion during level walking and compared them to the CL limb.

We hypothesized that passive AP translation, as well as passive axial rotational laxity of the PCL insufficient knees, will be increased compared to the CL knees, but that the range of tibiofemoral axial rotation and AP-translation of the PCL insufficient knees will not be increased compared to the CL knees during level walking.

## Materials and methods

### Patients

9 patients with isolated PCL insufficiency or PCL insufficiency combined with a PLC insufficiency were recruited. Inclusion criteria were a confirmed PCL insufficiency through clinical examination, magnetic resonance imaging and stress radiography. In stress radiography, a PCL insufficiency was determined through an increased posterior tibiofemoral translation of at least 5 mm compared to the CL knee. Further inclusion criteria were a healthy CL knee and adulthood. Exclusion criteria were previous PCL reconstruction, PCL avulsion fracture, body mass index (BMI) ≥ 35, ligament instability or previous surgery as well as flexion–extension limitations in the CL knee, osteoarthritis ≥ grade II by Kellgren and Lawrence, infection in one knee and pregnancy. All patients had a chronic PCL insufficiency with a history of knee injury on the PCL insufficient side more than three months ago. The demographic data of the analysed patient cohort is shown in Table 1**.** The study was approved by the local ethics committee (Nr: EA2/141/14). All subjects provided written informed consent prior to participation and were properly informed about the different measurement procedures.

**Table 1:** Demographic data of the analysed patients.

Patient	Gender	Age	Location	BMI	Injury type
1	m	45	Right	27.3	PCL
2	m	38	Right	24.8	PCL
3	m	43	Left	29.3	PCL
4	w	24	Left	19.8	PCL + PLC
5	m	35	Left	28.0	PCL
6	m	28	Right	28.1	PCL
7	m	34	Right	28.0	PCL+ PLC
8	m	38	Right	29.1	PCL + PLC
9	m	46	Right	34.2	PCL
Mean	–	36.78	–	27.6	–
SD	–	7.46	–	3.84	–

*PCL* Posterior cruciate ligament, *PLC* Posterolateral corner.

### Functional assessment of knee joint kinematics

#### Measurement of passive knee joint laxity

##### Passive rotational knee joint laxity

A certified and evaluated rotometer device (Berlin CERT, certification number: Z-11-131-MP) was used for accurate and objective measurement of passive rotational laxity. The device allowed axial internal and external rotation of the tibiofemoral joint to be performed at a knee flexion angle range of 0°–90°. Specific details on technical specifications as well as measurement procedure have been described previously^29,30^.

**Figure 1:**
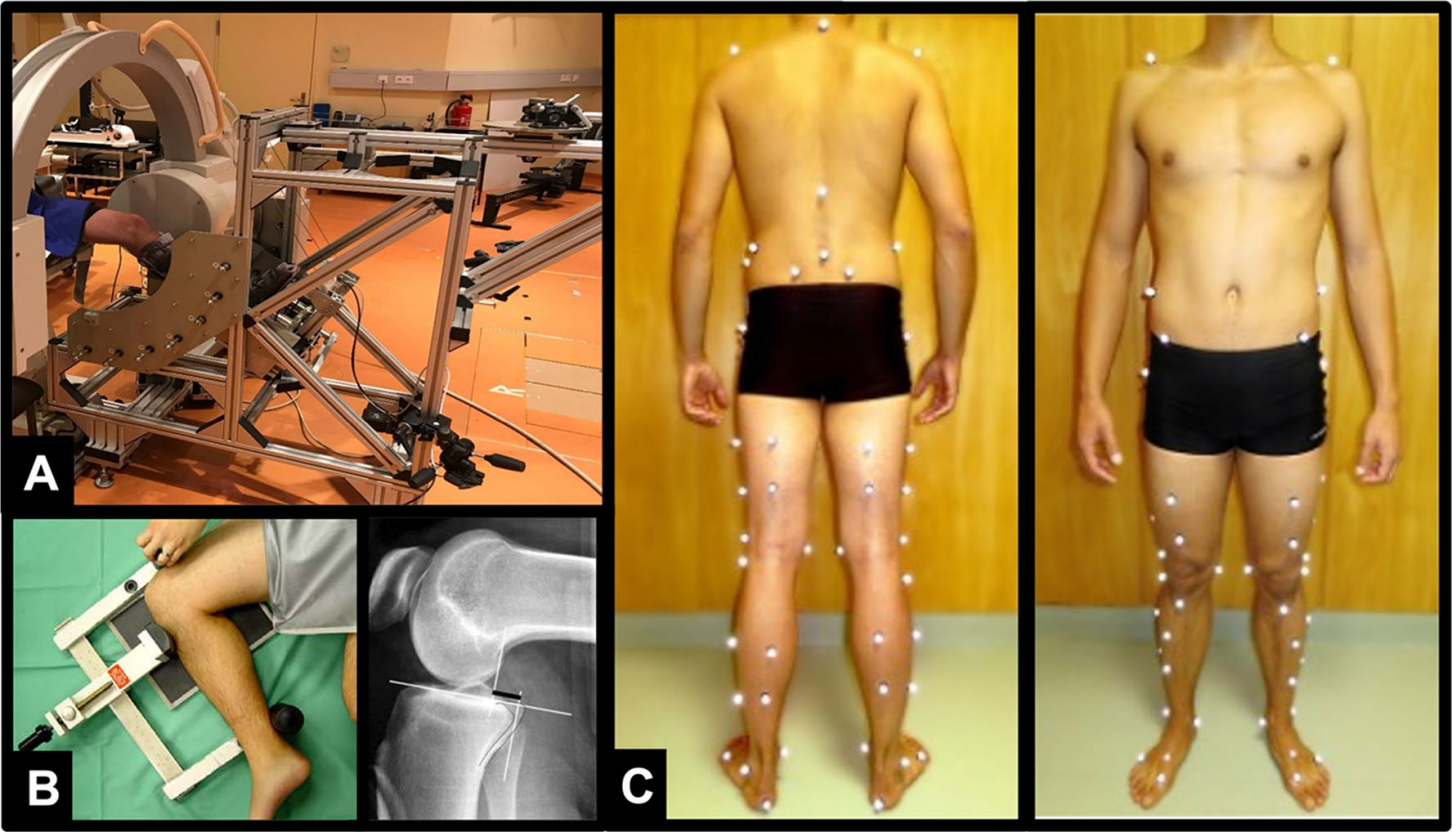
Measurement setup to assess passive knee joint rotation (**A**), passive posterior translation with stress radiography (**B**), and marker set of motion gait analysis to determine in-gait knee joint rotation and translation (**C**).

In the present study, a maximum internal and external torque of 3 Nm was used to simulate the conditions used in the clinic during the analysis of passive tibiofemoral rotation. The patients were instructed to relax their leg muscles during the measurements. The applied torque of 3 Nm was deliberately chosen because it has been used in several in-vitro studies to determine tibiofemoral rotational laxity and allows gentle testing of tibiofemoral rotational stability, corresponding to the force applied in the clinical dial test^31–33^. A C-arm fluoroscope (Pulsera BV, Philips, Amsterdam, Netherlands) was positioned at the level of the knee with the center of the knee as near as possible to the center of the image intensifier (Fig. 1A). The fluoroscopic system was calibrated prior to each measurement to correct for image distortion^34^. Fluoroscopic images were collected during the complete axial rotation cycle at a frequency of 3 Hz. Use of X-rays (Fluoroscopy) on the subjects was approved by the German Federal Office for Radiation Protection (Bundesamt für Strahlenschutz, Approval Number: Z5-22462/2-2014-096).

Measurements were performed at 30°, 60° and 90° of knee flexion. At 30°, the structures of the posterolateral corner (PLC) inhibit external tibiofemoral rotation^35^. With increased knee flexion, the tension in the PCL increases, leading to a higher contribution of the PCL for rotational stabilization^5,36^.

##### Passive translational knee joint laxity by means of stress radiography

With the device according to Scheuba et al. (Telos GmbH, Marburg, Germany) stress images of both legs were assessed to quantify the posterior and anterior tibial displacement in a side-by-side comparison (Fig. 1B). Within the device, the leg is fixed in 90° knee flexion, while the tibia can be pressed into the anterior or posterior drawer with a defined force of 150 N. For assessment of the PCL function, the knee was flexed at 90° while the pressure was exerted at the level of the tibial tuberosity. Four X-ray images for posterior and anterior drawer of both legs were generated to determine the side-to-side difference^16^. Assessment of the anterior drawer is important to exclude a fixed posterior drawer^37^. Subsequently, the Jacobsen technique was used to quantify the posterior and anterior drawer^38^.

#### Measurement of active knee joint kinematics during level walking

Level walking was chosen as an active movement as it is an important daily activity that can be conducted properly by patients with PCL insufficiency. The tibiofemoral kinematics during level walking were assessed by a set of 59 reflective markers^39^ attached to each patient’s body whose positions were tracked at 120 Hz using an infrared optical motion capture system (10 T20S cameras, Vicon, Oxford, United Kingdom), while the patients performed 10 repetitions of self-paced walking (Fig. 1C). For the quantification of relative tibiofemoral rotation and translation, an approach based on a combination of the optimal common shape technique (OCST), the symmetrical axis of rotation approach (SARA) and the symmetrical center of rotation estimation (SCoRE) was used to assess skeletal kinematics^39,40^ whose approach is precisely explained in Boeth et al.^41^.

### Data analyses

#### Fluoroscopic analyses and quantification of skeletal tibiofemoral rotation

In order to assess the kinematics of the knee joint, an analysis-by-synthesis approach was adapted to estimate the motion of skeletal joint structures from fluoroscopic images. An advantage of radiographic imaging is that it provides projections of the bony structures such that analysis is not impaired by soft-tissue motion as in, e.g., optical motion capture.

#### Pseudo computer tomography (pseudo-CT) synthesis

The subject-specific 3D anatomy of the knee bones was determined via magnetic resonance imaging (MRI). In order to extract the shape, we employed a convolutional neural network with U-net architecture^42^ in combination with statistical shape models (SSM)^43^ as anatomical prior for regularization.

To augment the subject-specific shape model with electron density information we employed an atlas-based approach in which we transferred a population-average density obtained from a pre-trained statistical shape and intensity model (SSIM). In particular, we computed a low-distortion map between the source and target volume in terms of differential coordinates^44^.

#### *3D*+ *t reconstruction*

**Figure 2:**
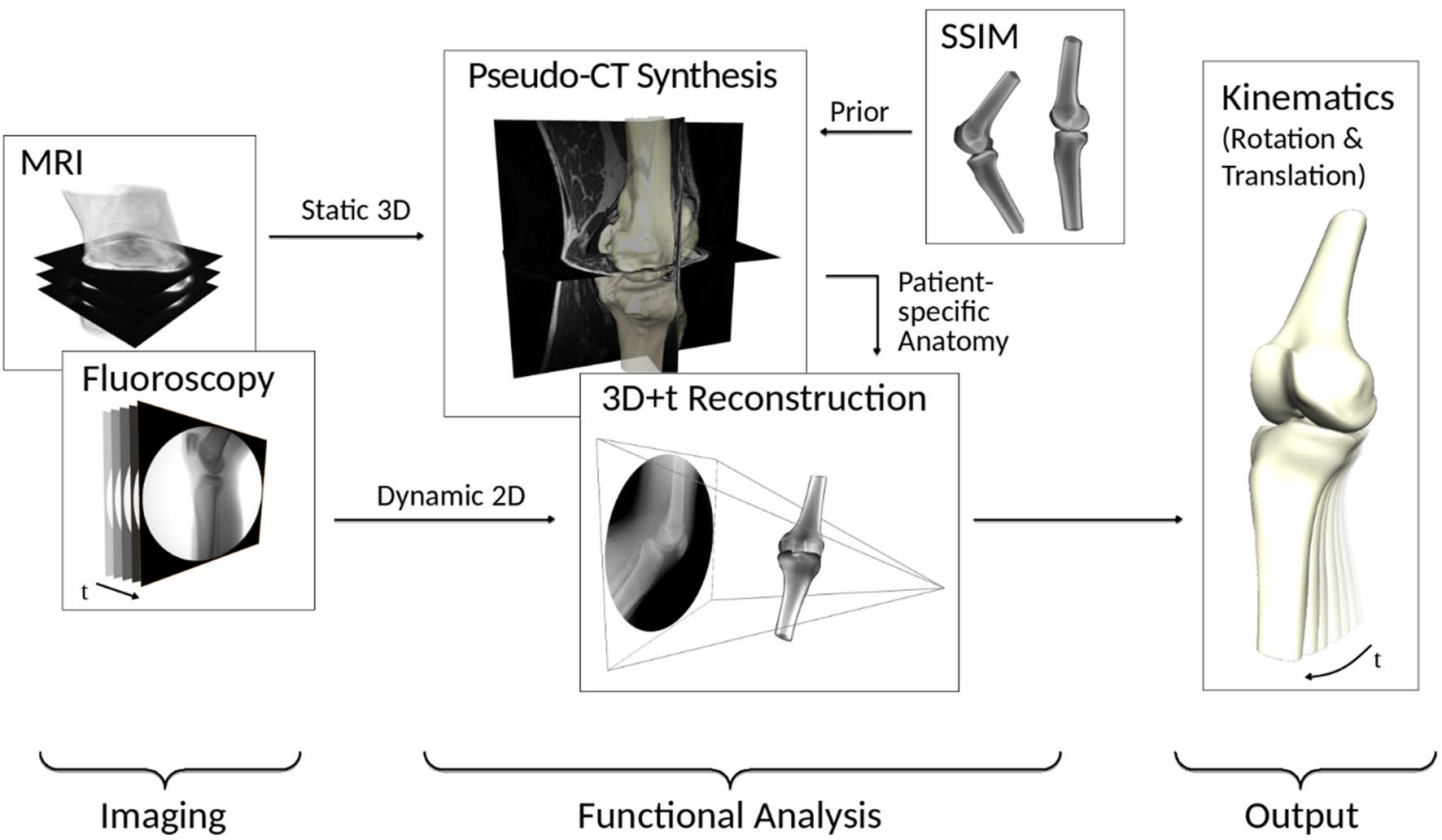
Determination of kinematic in vivo data from MRI and fluoroscopy data.

The transformation, i.e. position and orientation, of the derived shape and appearance model within the fluoroscopic imaging setup can then be determined using an image-based 3D/2D registration^45^, which has been shown to provide sub-millimeter and sub-degree accuracies for in-plane translation and rotation, respectively^46^. The transformation of the model is determined by iteratively optimizing the similarity measure, viz. normalized gradient fields^47^, calculated between a virtual X-ray image generated for the current estimate and the fluoroscopic image. While we performed manual initialization for the first frame of each fluoroscopic sequence, we could propagate it to the subsequent ones using extrapolation. To this end, we employed the group structure of the space of transformations to construct a smooth path interpolating the previous two transformations based on the exponential map. This approach led to an improved temporal coherence and numerical stability in our experiments. An overview of the registration pipeline is shown in Fig. 2.

The following specifications were considered during MRI scans: Proton density-weighted MRI scans, slice thickness 0.6 mm; voxel size 0.46 mm × 0.46 mm × 0.6 mm; time to repeat (TR) 1200 ms, time to echo (TE) 36 ms; flip angle 120°; 160 slices.

Torque-rotation curves were constructed for every measurement time point using the applied axial torque and the calculated axial rotation from the fluoroscopic sequences. The peak rotations at ± 3 Nm were used as a measure of internal and external rotational laxity. To correct for the effect of each subject’s natural knee rotation angle, the neutral reference rotation for each subject was determined as the average angle at which zero resistance to rotation was observed (taking rotation in both the internal and external directions into consideration). These neutral reference positions were then used for group-wise analyses.

#### Stress radiography

The posterior and anterior drawer of the PCL insufficient and CL knee joint during stress radiography were quantified by using the Jacobsen technique^38^. This technique uses peripheral bony landmarks to determine tibial displacement relative to the femur. First, a straight line is drawn along the medial tibial plateau. Two perpendicular lines are drawn onto the tibial plateau, starting from the center of the most posterior medial and lateral contours of the femoral condyle and the center of the most posterior edges of the medial and lateral tibial plateau. The distance between these perpendiculars represents the total posterior displacement. It is measured in millimeters. Physiologically, there is a correspondence between the tibial and femoral perpendiculars in the transverse plane^16^. The measurement was performed independently by two different examiners. Subsequently, the mean of both results was determined.

#### Tibiofemoral rotation and translation during walking

The 3D tibiofemoral motion data during self-paced walking were split into multiple repetitions of individual gait cycles^48^. For each kinematic variable, 101 discrete points according to 0–100% (heel strike to heel strike) of the gait cycle were extracted at 1% intervals using interpolation. The range of motion (RoM) for the rotation and the AP translation during each gait cycle was calculated as the difference between the minimum and maximum rotational and AP translational movement of the coordinate systems relative to one another. To determine group differences, each of the kinematic curves was averaged across trials for each patient as well as across all patients in each cohort.

### Statistical analysis

An a priori power analysis was performed for the passive rotational knee joint measurements based on a previous study using the same rotometer device for the analysis of passive rotational knee joint laxity^30^. Assuming a difference of 5° in passive rotational RoM between the injured and contralateral knees, a minimum sample size of 5 subjects was required to achieve a statistical significance of 0.05 with 80% power. The data was tested for normal distribution by Shapiro–Wilk-Test. For comparison of the PCL insufficient and the CL knee, paired t-tests for dependent samples were calculated with a significance level of 0.05. All statistical analyses were performed using IBM SPSS Statistics 25 (Chicago, Illinois, USA).

### Ethics approval and consent to participate

The study was approved by the local ethics committee (Ethikkommission der Charité-Universitätsmedizin Berlin, approval-Nr: EA2/141/14). Use of Fluoroscopy on the subjects was approved by the Bundesamt für Strahlungsschutz (Approval Number: Z5-22462/2-2010-003). All subjects provided written informed consent prior to participation and were properly informed about the different measurement procedures. All investigations were performed in accordance with relevant guidelines/regulations.

### Consent for publication

All subjects provided written consent for publication.

## Results

### Passive rotational knee joint laxity

**Figure 3:**
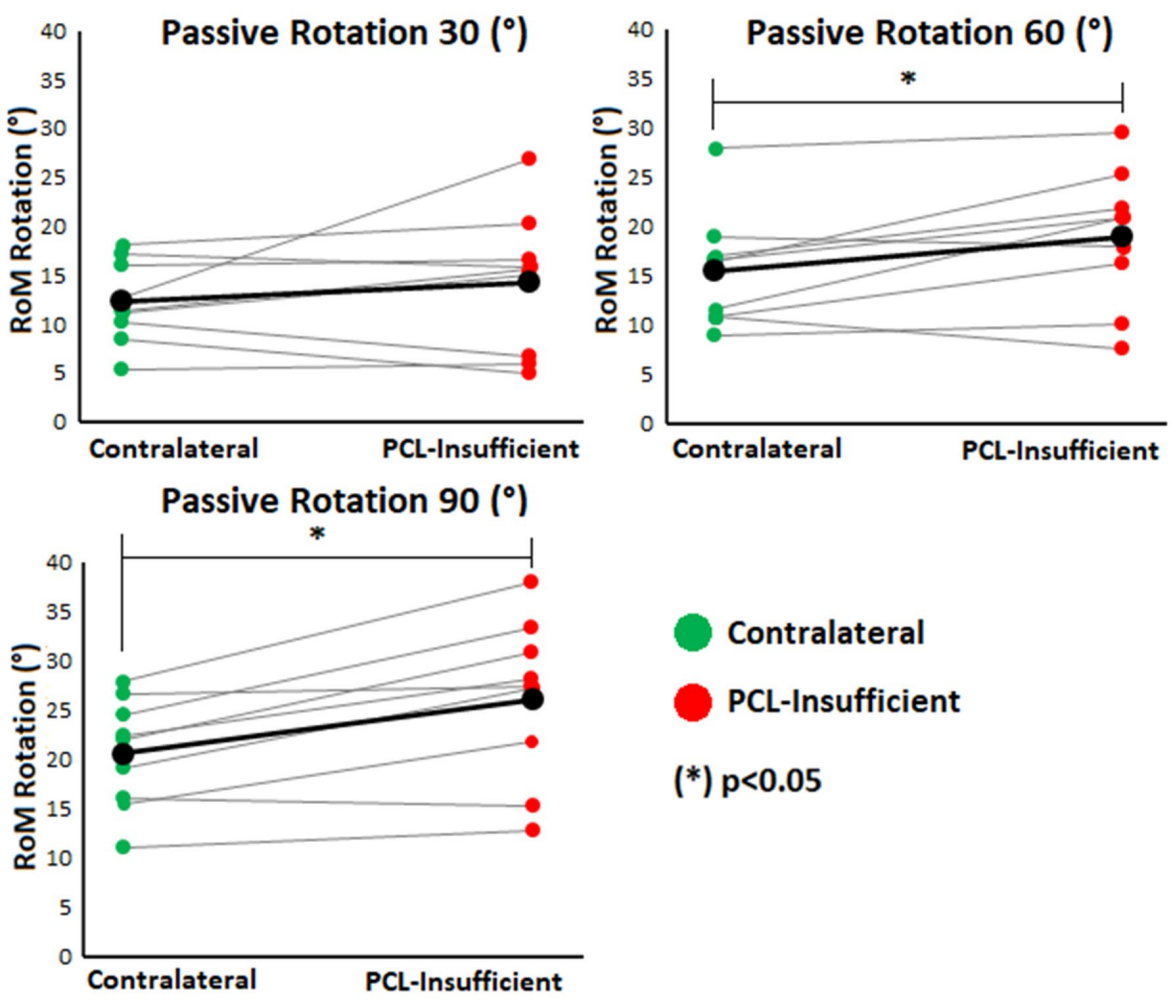
Passive rotational range of motion (RoM) of each knee joint at 30, 60 and 90° knee flexion.

Subjects with PCL insufficiency showed a significant increase in passive RoM at 90° and 60° of knee joint flexion compared to the CL knees (26.1 ± 8.2° vs. 20.6 ± 5.6° at 90° knee flexion, *p* < 0.01; 19.0 ± 6.9° vs. 15.5 ± 5.9° at 60° knee flexion, *p* = 0.04) (Fig. 3). At 30°of knee joint flexion, no significant increase in passive RoM was observed in the PCL insufficient knees in comparison to the CL knees (14.2 ± 7.3° vs. 12.3 ± 4.2°, *p* = 0.32).

### Passive translational knee joint laxity

The analysis of passive posterior translation knee joint laxity showed that subjects with PCL insufficiency had significantly increased values of 15.5 ± 5.9 mm at the PCL insufficient side compared to 3.7 ± 1.9 mm at the CL side (*p* < 0.01).

### Range of rotational and anterior–posterior tibiofemoral motion during level walking

The analysis of the range of anterior–posterior and rotational tibiofemoral motion during level walking conditions showed no significant differences in the AP translation of PCL insufficient knees compared to the CL knees (17.0 ± 5.4 mm vs. 16.1 ± 2.8 mm, *p* = 0.55).

For the rotational component during level walking, subjects with PCL insufficiency showed no significant differences of the PCL insufficient knees compared to the CL knees respectively (16.3 ± 3.7° vs. 15.2 ± 3.6°, *p* = 0.43). The self-paced walking speed of the analyzed patients was 1.15 ± 0,11 m/s (4.12 ± 0.4 km/h). The mean values for tibiofemoral AP translation and tibiofemoral rotation during the entire gait cycle are shown in the supplementary material.

### Knee joint extension-flexion angle analysis during level walking

No significant differences have been obtained for the range of flexion over the entire gait cycle, the maximum flexion angle, the maximum extension angle, the maximum extension angle at mid stance (10–30% of gait cycle) and the maximal flexion at initial swing phase (60–75%). A tendency for an increased maximum extension angle at mid stance (10–30% of gait cycle) in PCL insufficient patients compared to their CL side could be observed (*p* = 0.19).

Detailed values as shown in Table 2. The mean values for knee flexion–extension angles during the entire gait cycle are shown in the supplementary material.

**Table 2:** Analysis of knee flexion–extension angles during level walking.

	Contralateral	PCL insufficient	*p*-value
Range of flexion over entire gait cycle (°)	58.18	57.44	0.33
Maximum flexion angle (°)	61.37	60.74	0.73
Maxium extension angle (°)	3.20	3.30	0.89
Maximum extension at mid stance (10–30%) (°)	10.06	11.81	0.19
Maximum flexion at initial swing (60–75%) (°)	61.32	60.68	0.45

## Discussion

In this study of 9 PCL insufficient patients, we observed a significant increased passive knee joint laxity for the translational and the rotational components. However, there was no significant increased range of tibiofemoral motion in terms of AP translation or axial rotation in the PCL insufficient knees compared to the CL knees in the active level walking situation.

The analysis of the passive rotational laxity examined at 90° and 60° of knee flexion showed a significantly higher tibiofemoral rotational laxity of the PCL insufficient knee compared to the CL side. This result confirms the close connection between a diagnosed PCL insufficiency and an increased rotational laxity of the tibiofemoral joint. At 30° of knee flexion, the increase of the passive tibiofemoral rotational laxity on the PCL insufficient side was smaller and non-significant compared to 90° and 60° of knee flexion (Fig. 3). This is possibly due to the increased tension of the PCL from extension to mid-flexion^36,49^.

Although there is little evidence of this passive behavior in-vivo, several in-vitro studies have shown similar results although at different magnitudes of applied axial torque. Already in their in vitro study from 1975, Girgis et al. showed an average increased tibiofemoral external rotation of 8° after resection of the PCL^27^. This increment in external rotation could be observed only when the cadaver knees were flexed. In the study conducted by Sekiya et al. a mean increase in tibiofemoral external rotation of 6.7° was observed after comparison to the intact joint at 90° of knee flexion^5^. Bae et al. showed an increment of 8° in external rotation in the analysed cadaveric knees with resected PCL and an applied torque of 6 Nm as well^50^. Under application of simulated ischiocrural and quadriceps femoris muscle forces to simulate an in-vivo situation an increased passive tibiofemoral laxity in external rotation was found in a set of cadaveric knees with resected PCL at 90° of knee flexion by the group of Gill and colleagues^4^. Moreover, three of the analysed patients had an additionally diagnosed PLC insufficiency, which leads to an increased laxity in tibiofemoral external rotation, as well^6^.

Additionally to the increase in passive rotational laxity of the PCL insufficient knee, the subjects showed an increase of passive posterior translation laxity of the PCL insufficient knees compared to the CL knees. This result was expected as it is well studied that a PCL insufficiency leads to an increase in passive posterior translation laxity of the affected knee^8^.

**Figure 4:**
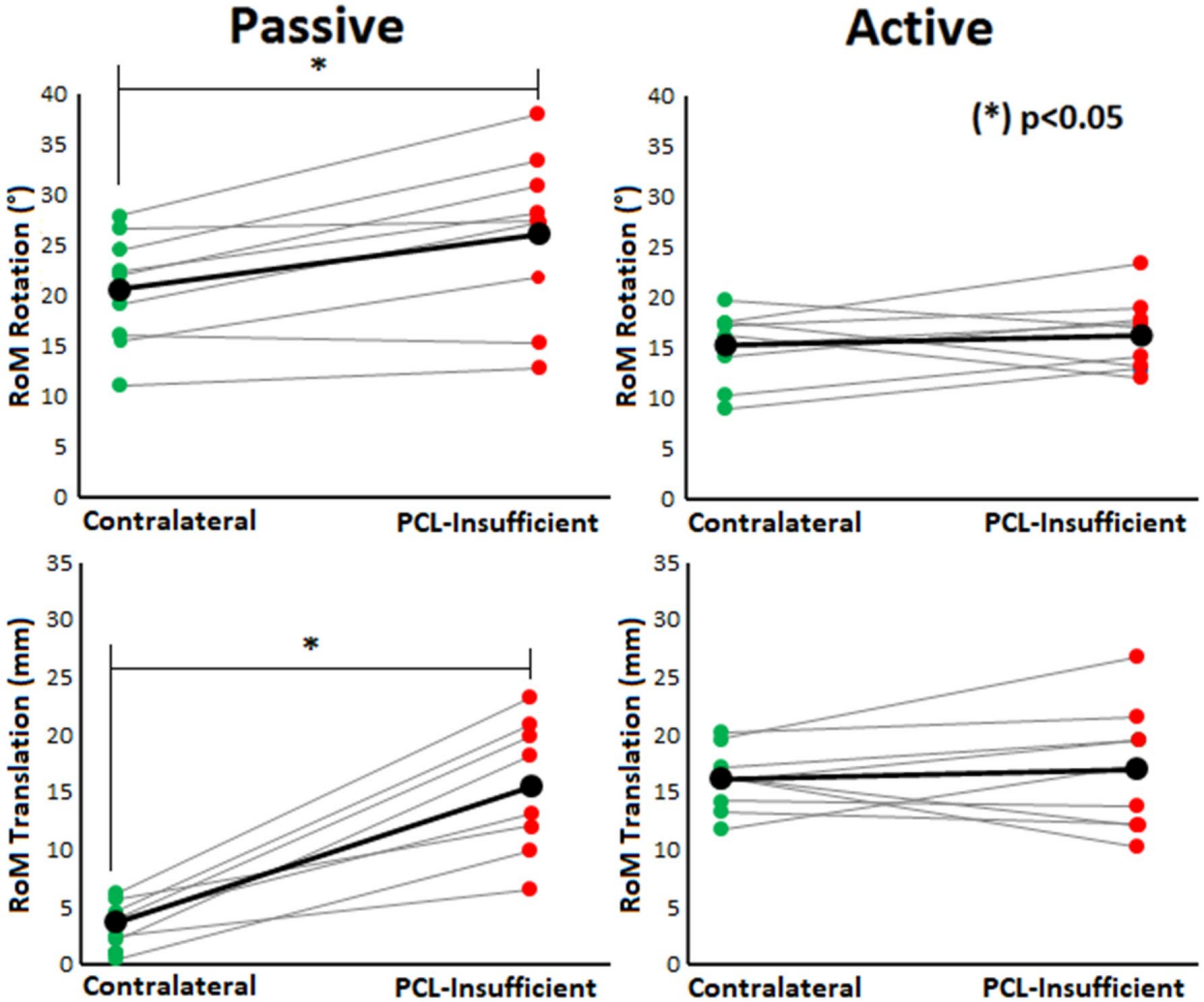
Comparison of passive rotational (at 90° of knee flexion) and passive translational RoM to active rotational and translational RoM during level walking. Significant differences between PCL insufficient and CL knees were found only during passive conditions. Range of Motion (RoM); Posterior cruciate ligament (PCL); Contralateral (CL).

As mentioned above, the results of the present study showed no significant increase in range of motion of tibiofemoral AP translation in the PCL insufficient knees in comparison to the CL knees during level walking conditions (Fig. 4). Regarding the axial rotation range of tibiofemoral motion, there were also no statistically significant differences found between PCL insufficient and CL knees during level walking conditions.

That patients with PCL insufficiency have no increased range of translation range of tibiofemoral motion during level walking could have multiple reasons. A reduction can be achieved during muscle activation, like contraction of the quadriceps muscles^25,51^. During the stand phase, an increased activation of the quadriceps muscles contributes to a reduction of the PCL loading^52^. Along the same line, an early activation of the gastrocnemius-soleus-complex has been observed in patients with PCL insufficiency^25,53^. On the other hand, changes in gait patterns contribute to compensation of passive laxity as well. Previous studies have shown an increased external rotation of the tibia during active movements in PCL insufficient subjects^18^. Reduced internal rotation of the tibia during the complete flexion range compared to the CL knees have been also observed with magnetic resonance imaging during single-leg lunge^54^. Earlier in-vitro studies have shown that the external rotation of the tibia with intact secondary structures contributes to the reduction of the dorsal subluxation^55^. Such changes in knee kinematics can lead to an incongruent position between joint contact areas and possibly to cartilage changes on the femoral medial condyle. During gait analysis conducted in patients with confirmed PCL insufficiency, the group of Orita and colleagues showed no increase in AP translation^18^, even a reduction of approximately 0.7 cm during terminal stance compared to a control group. Additionally, a significantly reduced flexion, as well as a slightly increased axial external rotation at 3–11 and 85–96% of the gait cycle and reduced peak axial internal rotation, was observed in the same study^18^. Goyal et al. found also no differences between PCL insufficient knees in comparison to PCL intact knees in their study where they analysed knee kinematics during level walking through Dynamic Stereo X-Ray in three patients with grade II PCL insufficiency. Nevertheless, they observed altered knee kinematics in the PCL insufficient knees compared to the PCL intact knees during running and stair ascent^22^. During analysis of the changes in gait parameters in patients with PCL insufficiency, the group of Fontboté et al. found no significant differences in knee flexion after comparison to a healthy control group, which corresponds with our data^21^. During assessment of subjective knee functionality with application of a “Flandry self-assessment of knee function” clinical questionnaire, Hopper et al. observed a correlation between high Flandy-Score and maximal extension moment as well as maximal knee extension during mid stance, which led them to the conclusion that PCL insufficient patients developed strategies to reduced knee loading during gait^56^.

Our results showed a tendency of a decreased extension at mid stance of the PCL insufficient knees compared the contralateral knees. Fleming et al.^57^ observed in patients with chronic PCL insufficiency a slightly bent knee during walking in order to reduce a terminal hyper extension and external rotation of the tibia due to dorsal subluxation of the lateral tibia plateau. Similar to our results, Yu et al. observed decreased knee extension angles in the PCL insufficient knees compared to the PCL intact knees during the terminal stance phase^26^. Perry et al.^58^ described four possible causes for inadequate knee extension during gait: flexion contraction, reduced hamstrings muscle activation, soleus-gastrocnemius insufficiency and quadriceps weakness. Tibonne et al.^25^ showed evidence of quadriceps insufficiency in patients with PCL insufficiency. Hopper et al.^56^ reported about weak knee extensors in a similar cohort compared to a healthy control group. The mentioned dorsal subluxation of the tibia during posterior instability leads to a forward displacement of the contact area between femur and tibia, which results in a reduction of the lever support of the quadriceps on the tibia during knee extension^59^. The limited flexion–extension degree in the PCL insufficient knee joint and the consequent limited rolling-sliding movement pattern could also contribute to a reduction of the AP translation during dynamic activities.

This study has some limitations. One limitation is the small sample size. The relatively low number of subjects is due to the fact that PCL insufficiency is a rare pathology and thus a general limitation in the research field of PCL insufficiency. However, the number of knee joints analysed in this study is comparable to several cadaveric and in vivo studies that successfully evaluated biomechanical properties of a PCL insufficiency^18,22^. Another major limitation is the lack of a separate healthy control group. The reason is ethical concerns regarding the use of the fluoroscope device on healthy subjects. Possible changes of the knee joint kinematics on the CL knees can therefore not be excluded. On the other hand, in a study conducted by Kozanek et al.^60^ on patients with one-sided PCL insufficiency during the performance of a knee bend until 90° of flexion, no significant differences were found between the CL knee on the PCL insufficient subjects and an additional healthy control group. Furthermore, we did not evaluate passive varus and valgus laxity, due to the fact, that PCL insufficiency has a minor effect on varus and valgus laxity of the knee joint^7^ and additional X-rays with an unjustified radiation dose would have been necessary. Regarding the measurements techniques used it is important to mention the reduced accuracy of the marker-based technique used for the active measurements compared to the fluoroscopy technique used for the passive measurements. However, it is important to highlight the reduction of around 50% on skin marker artifacts after application of the OCST approach during marker-based measurements^40^. Future dynamic fluoroscopy measurements on PCL deficient patients are currently planned to improve the aforementioned disadvantage.

## Conclusion

The present study contributes to the general understanding of joint kinematics in the passive and active state in patients with PCL insufficiency. The analysed patients showed substantially increased laxity, not only in passive tibiofemoral translation but also in passive tibiofemoral rotation compared to the CL knees. On the contrary, no significant differences were observed for the rotational and translational assessments of tibiofemoral range of motion during level walking conditions, which indicates that this multiplanar increased knee joint laxity in the passive situation could be compensated during level walking conditions.

Changed kinematics in PCL insufficient knees could present potential risk factors for progressive cartilage degeneration^19^. Future work should therefore include the analysis of progressive changes in knee joint kinematics after PCL insufficiency and PCL reconstruction. Also, the effect of quadriceps force training on gait patterns in PCL insufficient patients should be analysed. The inclusion of standardized, multidimensional, objective measurements for diagnosing rotational and translational laxity in clinical assessments should be considered.

## Supplementary Information

Supplementary Information.

## Data Availability

The data are available from the corresponding author upon reasonable request.

## Abbreviations

PCL: Posterior cruciate ligament
PLC: Posterolateral corner
AP: Anterior–posterior
OCST: Optimal common shape technique
SARA: Symmetrical axis of rotation approach
SCoRE: Symmetrical center of rotation estimation
Pseudo-CT: Pseudo computer tomography
3D/2D: Three dimensional/two dimensional
MRI: Magnetic resonance imaging
SSM: Statistical shape models
SSIM: Statistical shape and intensity model
TR: Time to repeat
TE: Time to echo
PD: Posterior drawer
AD: Anterior drawer
RoM: Range of motion
CL: Contralateral
