# Supplementary material for: PCL insufficient patients with increased translational and rotational passive knee joint laxity have no increased range of anterior–posterior and rotational tibiofemoral motion during level walking
Source: Sci Rep. 2022 Aug 2;12:13232. doi: 10.1038/s41598-022-17328-3 (PMC9345965; doi:10.1038/s41598-022-17328-3)
Supplement: Supplementary file 1 — Supplementary Information. [file 41598_2022_17328_MOESM1_ESM.pdf]

Supplementary material

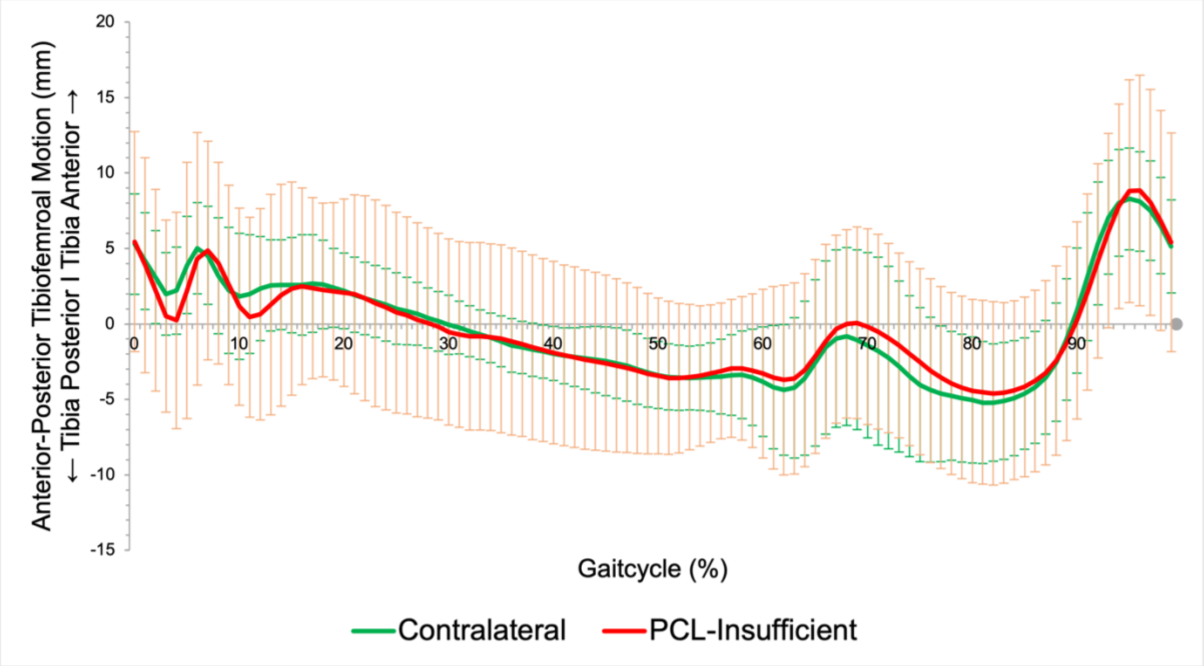

Supplementary figure 1. Anterior-posterior tibiofemoral motion (mm) of PCL insufficient knees (red) and contralateral knees (green) during the entire gait cycle. Values are mean ± standard deviation.

Posterior cruciate ligament (PCL).

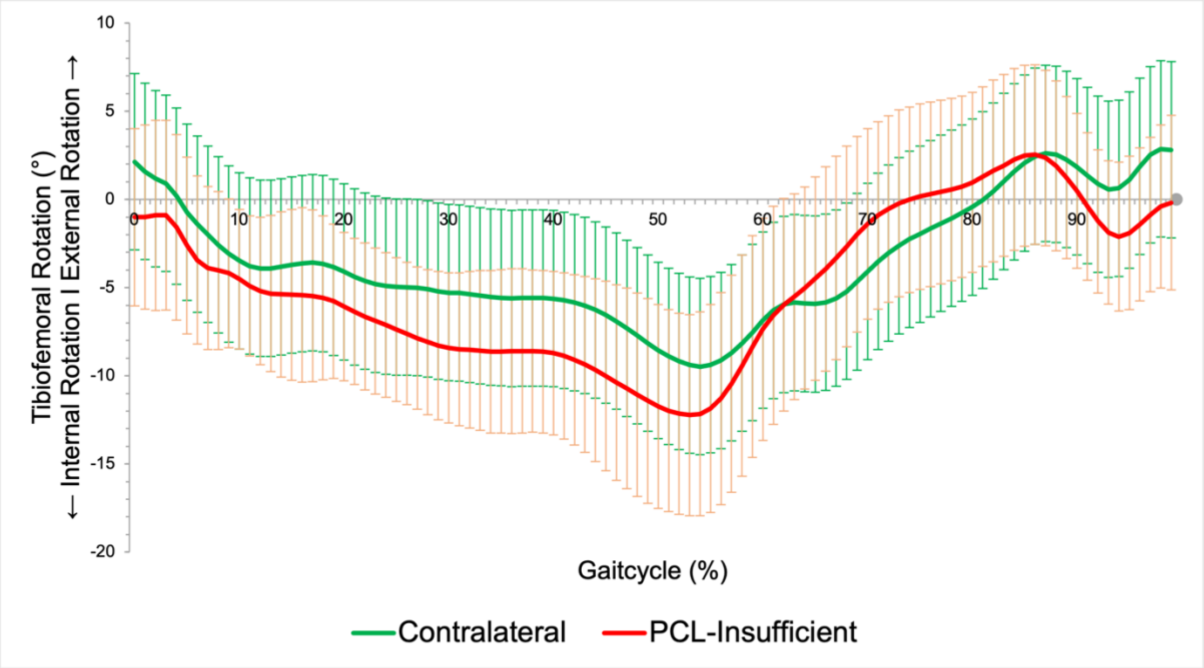

Supplementary figure 2. Tibiofemoral rotation (°) of PCL insufficient knees (red) and contralateral knees (green) during the entire gait cycle. Values are mean ± standard deviation. Posterior cruciate ligament (PCL).

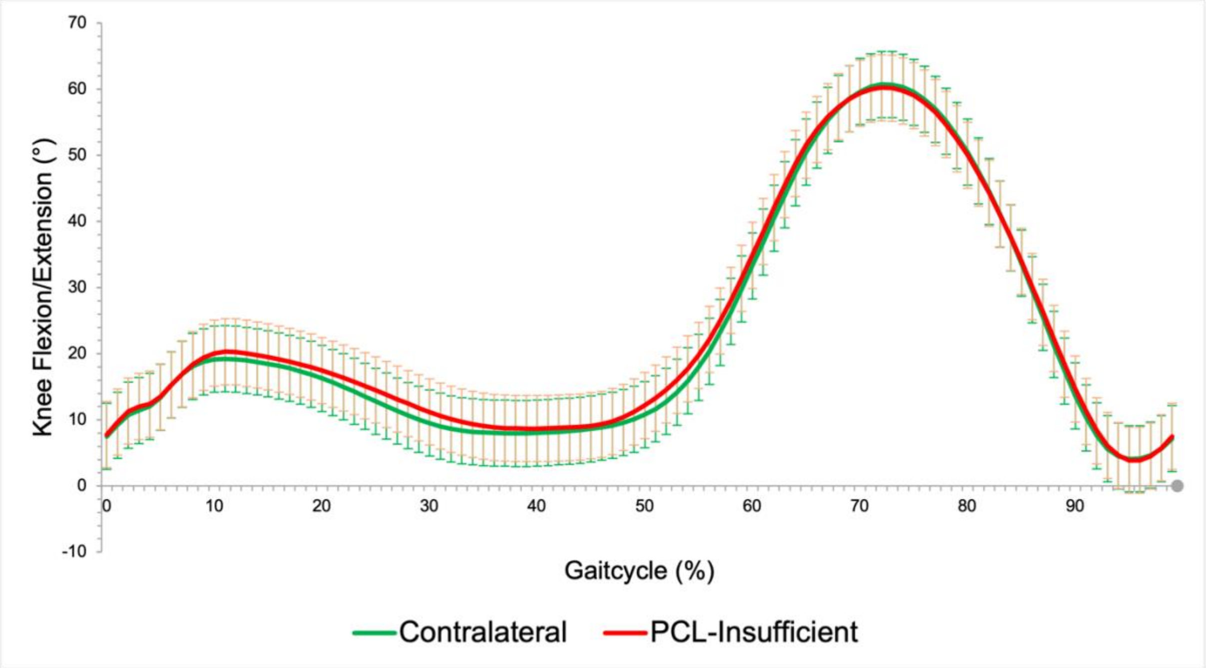

Supplementary figure 3. Knee Flexion/Extension (°) of PCL insufficient knees (red) and contralateral knees (green) during the entire gait cycle. Values are mean ± standard deviation. Posterior cruciate ligament (PCL).
